# Schizophrenia or Atypical Lupus Erythematosus with Predominant Psychiatric Manifestations over 25 Years: Case Analysis and Review

**DOI:** 10.3389/fpsyt.2017.00131

**Published:** 2017-07-27

**Authors:** Axel Mack, Christiane Pfeiffer, E. Marion Schneider, Karl Bechter

**Affiliations:** ^1^Department of Psychiatry and Psychotherapy II, University Ulm/Bezirkskrankenhaus Guenzburg, Guenzburg, Germany; ^2^Department of Dermatology and Allergology, University Hospital Ulm, Ulm, Germany; ^3^Sektion Experimentelle Anaesthesiologie, University Hospital Ulm, Ulm, Germany

**Keywords:** mild encephalitis, neuroinflammation, chronic schizophrenia, autoimmune encephalitis, autoimmune diseases, neuropsychiatric lupus erythematosus, lupus erythematosus, systemic

## Abstract

We observed a case over 25 years of relapsing–remitting schizophrenic spectrum disorder, varying regarding the main symptomatology between more depressive or more schizoaffective or rather typical schizophrenic syndrome. Diseased phases were repeatedly accompanied by minor skin lesions, which were initially classified as mixed tissue disorder. Psychotic phases were waxing–waning over years. During one later relapse, skin involvement was severe, classified to likely represent an allergic reaction to psychopharmaca; this generalized exanthema remitted rapidly with cortisone treatment and azathioprine. Under continued azathioprine and low dose neuroleptics, the patient remitted completely, appearing psychiatrically healthy for 16 years. When azathioprine was set off due to pregnancy, an extraordinary severe relapse of schizophrenia like psychosis accompanied by most severe skin lesions developed within a few weeks, then requiring 2 years of psychiatric inpatient treatment. Finally, a diagnosis of systemic lupus erythematodes plus neuropsychiatric lupus was made. A single CSF sample in 2013 showed suspicious biomarkers, matching with CSF cytokine profiling in schizophrenic and affective spectrum disorder patients and indicated mild neuroinflammation. Complex immune suppressive treatment was reinitiated short after relapse, but was only partially successful. However, surprisingly the psychosis and skin lesions remitted (in parallel) when belimumab was given (add-on). The very details of this complicated, long-term disease course are discussed also with regard to general ideas, in particular with respect to the question if this case of seemingly comorbid schizophrenia with minor autoimmunity signs represented a case of one emerging autoimmune disorder with variant manifestations systemically and within the CNS, though atypically with predominant appearance as a schizophrenia spectrum disorder.

## Introduction

Schizophrenia is understood as a partly heritable brain disease, recent interesting findings showing alleles of the complement component C4 genes play a role in both in the CNS and the immune system (1). Autoimmune diseases and severe infections are associated with a later increased risk of schizophrenia and other psychiatric disorders (2, 3). The relative risk of schizophrenia for an individual with a history of autoimmune disease in themselves or in their family is elevated by about 45%. Inversely schizophrenia is associated with a nearly 50% elevated lifetime prevalence of autoimmune diseases (4).

We observed a case of a female patient with a 25-year psychiatric history, her disease beginning at the age of 24. As the medical records show, the diagnosis was changing several times, from depression (bipolar disorder hypothesized) to schizoaffective to schizophrenic disorder, this was the predominating diagnosis over disease cause. Besides psychiatric process, the disease course in this patient was atypical, as her psychotic phases were often accompanied by appearance of minor non-specific skin lesions on the trunk and limbs. It has been suggested that there might be an autoimmune process; however, she did not fulfill any criteria for a defined autoimmune disorder. During one particular psychotic relapse she developed severe skin efflorescences, which were treated with cortisone and later azathioprine. Under this regimen skin improved rapidly and surprisingly after this particular inpatient treatment, the patient remained psychiatrically well with few exceptions over many years. Sixteen years later, this patient became pregnant and azathioprine had been set off because of possible teratogenic side effects. Soon, after discontinuing azathioprine, she rapidly relapsed and for the first time the criteria of an established autoimmune disorder, systemic lupus erythematosus (SLE), were fulfilled and could be even extended to neuropsychiatric lupus erythematosus (NPSLE).

The question discussed in this single case, is whether the patient suffered from the two unrelated disorders of schizophrenia and SLE, or an atypical course of a not clearly defined autoimmune disorder with early predominant psychiatric and late neuropsychiatric manifestations, with minor systemic manifestations and late fulfilling criteria of SLE/NPSLE.

## Background

In previous CSF studies, we accumulated evidence of minor neuroinflammation and immune activation in large subgroups of both schizophrenic and affective disorders (5). These findings were recently confirmed by others (6). In addition we recently found high CSF cytokines, especially at IL-8 in each patient (7), also increased CSF neopterin (8). All these findings supported the recently updated mild encephalitis (ME) hypothesis (9). Also epidemiological studies are well compatible with ME hypothesis, in that infections and autoimmune disorders are additive risk factors for a spectrum of severe psychiatric disorders (3). Discrete features of neuroinflammation are seen in a variety of CNS disorders, including degenerative diseases like Alzheimer’s disease, where neuroinflammation seems to represent a disease escalating factor (10). Most interesting is that in single case studies of acute psychosis, despite normal magnetic resonance imaging scans of the brain and normal CSF and without detection of CNS autoantibodies, there was nevertheless proof of mild neuroinflammation in the cortex biopsy (11). These findings point to the difficulties in detecting mild inflammatory processes and show the limitations of available diagnostic methods including CSF diagnostics (12).

The study was approved by the ethics committee of the University of Ulm (the patient gave written informed consent to publication) was to gain a better insight into possible relationships between a seemingly primary psychiatric disorder and a poorly defined autoimmune process. By using a careful retrospective analysis of an unusual case with a long-standing disease course and considerable available clinical material, the possibility of a unifying diagnosis over the disease course, against the established assumption of two separate disorders (schizophrenia and autoimmune disorder) was tested.

## Family History

Within the framework of the patient’s sixth hospitalization, the suspicious family history for both psychiatric and autoimmune disorders became apparent: both parents and the maternal grandmother suffered from a longtime depression with several psychiatric hospitalizations; the grandmother committed suicide. The patient’s only sibling has a schizophrenic disorder as well as Crohn’s disease.

## Retrospective Case Analysis of 25 Years

In 1989, a 24-year-old woman suffering from severe depression (ICD-10 F32.3) with predominant loss of interest, hypersomnia, feelings of guilt, and worthlessness and diminished ability to concentrate was hospitalized in our clinic for the first time. The physical findings were unremarkable except slight anemia, increased blood sedimentation rate and positive rheumatoid factor. Besides the psychiatric symptomatology no signs of autoimmunity were registered. The patient was treated successfully with a combination of pimozide, flupentixol, and amitriptyline and released after 3 months inpatient stay in good mental condition.

During 1990, now 25 years old, the patient again demonstrated emerging anxiety, thought disorder (derailment and thought blocking), disorganized speech and for the first time acoustic hallucinations (commenting voices) and initially catatonic stupor. In the required second psychiatric hospital stay she showed slight leukopenia and hypochromic microcytic anemia. One month after hospitalization some non-specific skin manifestations on her chest and back appeared. Internal and dermatological consultancies happened, tissue samples were made, but neither serological nor histopathological results could name the disorder. Now the differential diagnosis of collagenosis was considered and further blood testing was undertaken. For the first time, antinuclear antibodies (ANA) tested positive. Bone marrow examinations showing no hematological disorder, brain MRI exposed no cerebral pathology. There was no evidence of infection, electrolyte disturbances or metabolic derangements. Established criteria of a defined autoimmune disorder were not fulfilled and therefore no immune suppressive therapy was started. With a combination of bromperidol and clorazepate, she distanced from productive psychotic content. During the cause, more depressive symptoms developed, tranylcypromine were added. The patient was dismissed in acceptable mental status.

**Figure 1:**
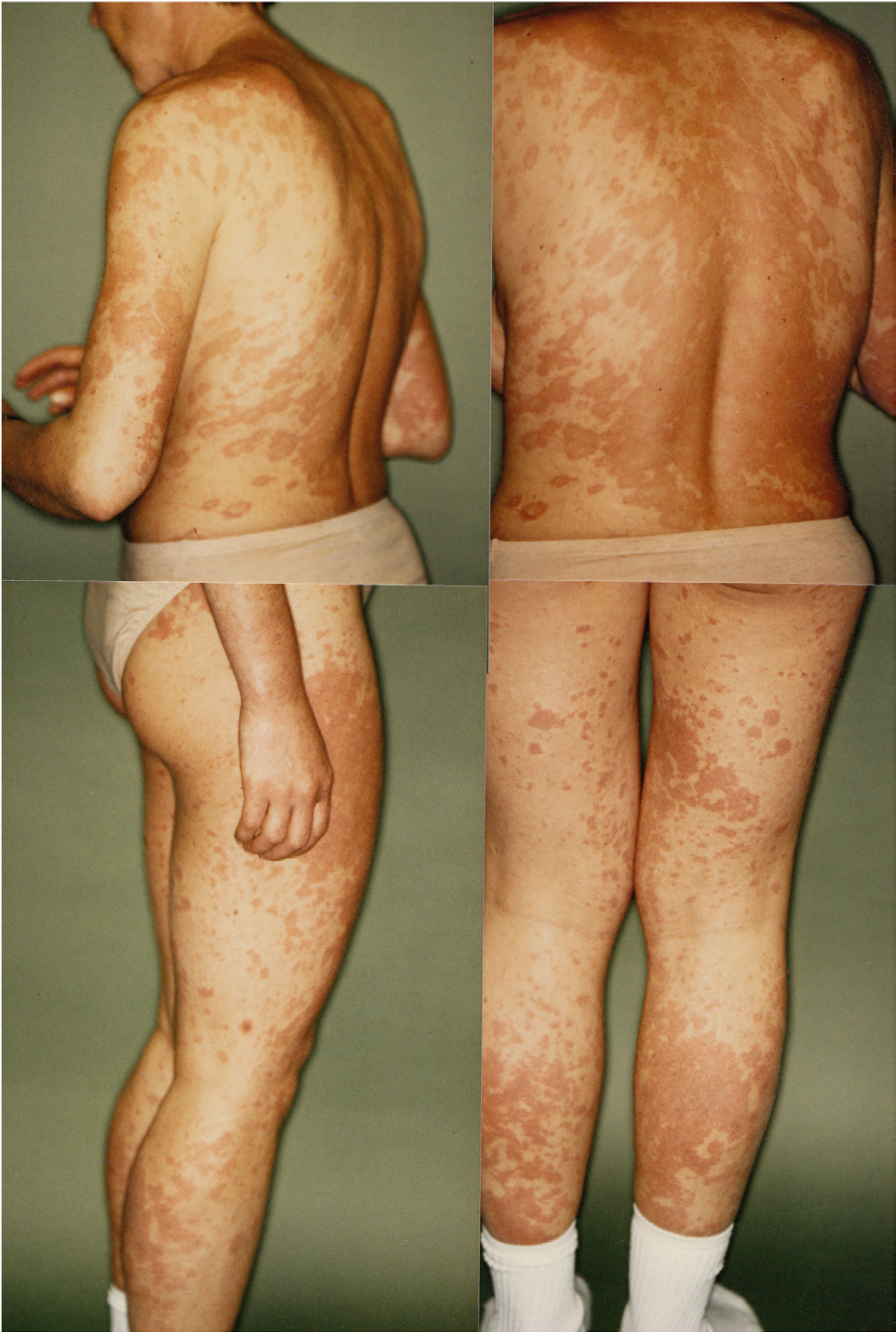
Skin manifestations of the patient in April 1993, hypothesized as Stevens–Johnson Syndrome.

In 1993, a third severe relapse occurred requiring hospitalization again. Main symptoms were restlessness, anxiety and extensive productive psychotic symptoms. One month after hospitalization, additional symptoms of severe maculopapular exanthema on the trunk and limbs appeared. This was diagnosed as a Stevens–Johnson syndrome and thought to be caused by the psychopharmacological drugs, propyphenazone ergotamine or acetylsalicylic acid (her own headache medication). The patient was transferred to the internal medicine department, where high-dose cortisone (100 mg/day) was initiated, 11 days later the exanthema had remitted, and patient was transferred back to the psychiatric ward. Cortisone was gradually phased out until the patient was discharged from the hospital. A few months later, in outpatient treatment the patient attempted suicide during a psychotic episode and was readmitted to the hospital. Clinical examination now demonstrated speckled exanthema on her chest, which was rapidly progressing, then confluating and spreading over the limbs (see Figure 1). Blood testing showed high ANA Titer and for the first time autoantibodies against Ro/SSA and La/SSB (see Table 1). In October 1993, an atypical collagenosis was hypothesized. Under this assumption, prednisolone was administered for over 6 months. During this period, skin and psychosis improved, though some symptoms continued to fluctuate.

**Table 1:** Exemplary serological immunoinflammatory markers of the patient over last 25 years.

		Treatment	ST (no. 1)	AMB	ST (no. 2)	ST (no. 3)	ST (no. 4)	AMB	ST (no. 5)	ST (no. 6)	AMB		ST (no. 7)	ST (no. 8)	ST (no. 9)
	
		Period of time	14.04.–23.06.1989	01.01.2000	15.02.–11.07.1990	11.03.–27.07.1990	20.08.1993–30.03.1994	20.02.1995	11.06.1996–12.06.1997	19.07.–25.09.2004	30.09.2008–30.04.2009	01.02.201020.02.2011	29.05.2012–03.05.2013	29.05.–13.12.2013	03.01.–06.05.2014
Laboratory marker		Reference range/unit													
Leukocytes		4.0–10.0 × 10^3^/μL	Normal	Normal	3.3	3.4	NDA	4.9	3.88	4.9	NDA	NDA	2.8	2.1	2,8
Hemoglobin		12.0–16.0 g/dl	Normal	Normal	11.2	12.2	NDA	17.3	12.3	13.0	NDA	NDA	11.0	11.3	11.5
ESR		<10/20 mm	38/76	29/64	26/54	NDA	33/65	56/60	18/55	19/46	14/33	14/35	NDA	NDA	17/39
Alpha 2 macroglobulin		7.4–12.6 rel%	High	NDA	NDA	NDA	NDA	9.1	8.1	NDA	NDA	NDA	NDA	13.2	NDA
Gamma-globulin		8.0–15.8 rel%	25.4	NDA	NDA	NDA	NDA	24.4	19.5	NDA	NDA	NDA	NDA	10.8	NDA

ANA		<1:80 Titer	Negative	Negative	NDA	NDA	1:10,240	1:2,400	1:10,000	1:1,200	1:9,600	NDA	NDA	1:2,560	1:2,560
	Anti-dsDNA	<10 or <100 U/ml	NDA	Negative	62	NDA	<40	<3	NDA	10	<2	<3	NDA	<0.5	NDA
	Anticentromere	<7 U*/*ml	NDA	NDA	NDA	NDA	Negative	NDA	NDA	Negative	NDA	NDA	NDA	<0.4	NDA
	Anti-histone AB	<25 U/ml	NDA	NDA	NDA	NDA	Negative	NDA	NDA	NDA	NDA	NDA	NDA	NDA	NDA
	Anti-Jo-1-AB	<7 U/ml	NDA	NDA	NDA	NDA	Negative	NDA	NDA	Negative	NDA	NDA	NDA	<0.3	NDA
	Anti-U1-RNP-AB	<25 U/ml	NDA	NDA	NDA	NDA	Negative	NDA	NDA	Negative	NDA	NDA	NDA	0.3	NDA
	Anti-Scl-70-AB	<25 U/ml	NDA	NDA	NDA	NDA	Negative	NDA	NDA	Negative	NDA	NDA	NDA	<0.4	NDA
	Anti-Smith-AB	<25 U/ml	NDA	NDA	NDA	NDA	Negative	NDA	NDA	Negative	NDA	NDA	NDA	0.3	NDA
	Anti-SSA/Ro-AB	Negative U/ml	NDA	NDA	NDA	NDA	182.5	NDA	NDA	4.4	NDA	NDA	NDA	>240.0	>240.0
	Anti-SSB/La-AB	Negative U/ml	NDA	NDA	NDA	NDA	117.5	NDA	NDA	2.0	NDA	NDA	NDA	2.5	NDA

Rheumatoid factor		<20 IU/ml	Positive	112	176	NDA	48.4	NDA	NDA	44	NDA	NDA	NDA	NDA	NDA
Anti-CCP-AB		<7 U/ml	NDA	NDA	NDA	NDA	NDA	NDA	NDA	NDA	NDA	NDA	NDA	NDA	NDA

aPL-AB	Lupus anticoagulant	<1.08 (Ratio)	NDA	NDA	NDA	NDA	NDA	NDA	NDA	NDA	NDA	NDA	NDA	NDA	NDA
	Anti-cardiolipin-AB	MPL-U/ml	NDA	Negative	NDA	NDA	NDA	NDA	NDA	NDA	NDA	NDA	NDA	NDA	Negative
	β_2_-Glycoprotein 1-AB	<7 U/ml	NDA	NDA	NDA	NDA	NDA	NDA	NDA	NDA	NDA	NDA	NDA	NDA	NDA
	Antiphosphatidylserine-AB	<10 IU/ml	NDA	NDA	NDA	NDA	NDA	NDA	NDA	NDA	NDA	NDA	NDA	NDA	NDA

Complement component 3		0.90–1.70 g/l	Low	Normal	NDA	NDA	1.13	NDA	NDA	NDA	NDA	NDA	NDA	0,89	1,03
Complement component 4		0.11–0.34 g/l	NDA	Normal	NDA	NDA	0.18	NDA	NDA	NDA	NDA	NDA	NDA	0.18	0,25

ANCAs	c-ANCA	1:<10	NDA	NDA	NDA	NDA	NDA	NDA	NDA	1:<10	NDA	NDA	NDA	NDA	NDA
	p-ANCA	1:<10	NDA	NDA	NDA	NDA	NDA	NDA	NDA	1:<10	NDA	NDA	NDA	NDA	NDA

*Laboratory marker and reference range/unit: AB, antibody; ANA, antinuclear antibody; CCP, (anti) citrullinated protein *antibodies*; DsDNA, double stranded deoxyribonucleic acid; ESR, erythrocyte sedimentation rate; aPL, antiphospolipid (antibody); ANCA, antineutrophil cytoplasmic antibodies*.

*Treatment: ST, stationary treatment (number of hospitalization in brackets); AMB, ambulatory treatment*.

*NDA, no data available; green, pathologically elevated; red, pathologically elevated*.

## Surprising Remission Under Azathioprine Treatment for Almost 16 Years

In the time period between 1994 and 1996 no severe problems were stated, according to the patient’s own evaluation “skin and mind came to rest.” However, in June 1996 another relapse of psychosis occurred: she presented initially symptoms of thought disorder, delusional ideas, paranoia, depersonalization and later acoustic hallucinations. Thioridazine, fluphenazine, and diazepam were added. In the course, fluphenazine was reduced and because of emerging comorbid depressive symptoms and affective instability, amitriptyline and later lithium was added. Because of suspected drug-induced leukopenia, fluphenazine was replaced by olanzapine. Skin eruptions on the trunk followed psychiatric symptoms about 5 months later. Another therapy trial with prednisolone was initiated, with worsening of her skin and mental status, so that treatment on a protected psychiatric ward became necessary. CSF examination ruled out any brain infection, however, did show unspecific inflammation. A diagnosis of mixed connective tissue disorder was then made. Azathioprine (Imurek^®^) was prescribed, starting in January 1997 and continued for nearly 16 years. About 2 weeks after the start of azathioprine, the exanthema regressed and prednisolone was tapered out. As the psychiatric symptoms gradually improved, with a complex psychopharmacological treatment including lithium, olanzapine, amitriptyline, the patient was discharged from hospital. Azathioprine was reduced from 100 to 50 mg future years. Because of her good mental health, olanzapine and amitriptyline were gradually reduced but maintained until 2004. Lithium was phased out in subsequent 2 years. Under this treatment the patient remained stable with regard to mental status and skin status for nearly 16 years (except for one exception in 2004). After release she was in psychiatric and sporadic rheumatologic supervision. Interim, she married a second time. She chose early retirement but was able to work part-time in a fashion store. The ambulatory protocols in this time noted occasionally social anxieties, but predominant an “emotionally and psychologically stable” status.

In 2004, a brief hospitalization became necessary, because of restlessness and affective tension, probably as a consequence of life events (suicide attempt of her mother and sister and the sisters diagnosis schizophrenic disorder). During inpatient stay, acoustic hallucinations were noted and quetiapine was added next to amitriptyline, olanzapine, and azathioprine. She was released after 2 months in good condition and was mentally stable for another 8 years.

## Severe Relapse after Discontinuance of Azathioprine

In 2012, because of pregnancy, the patient decided together with the gynecologist to stop azathioprine medication immediately, balancing the teratogenic potential versus the need for continuing azathioprine. Shortly after, the pregnancy was terminated at the request of the patient, but azathioprine was still left out. As a result, an unexpected and dramatic change in the overall health status of the patient reoccurred. Within 4 weeks the patient developed signs of restlessness, insomnia and mania, all symptoms rapidly deteriorating further to a full blown psychosis, and again requiring hospitalization in May 2012. She soon developed progressive disorganized behavior, thought disorder overall incoherence and paranoid ideation. The initial therapy was undergoing a continual adjustment, including benperidol (stopped because of side effects), later fluphenazine, quetiapine, asenapine, and because of depressive symptoms trimipramine and amitriptyline. Finally, a combination of risperidone, olanzapine, chlorprothixene, and valproate was given. Electroconvulsive therapy was tried. Besides psychotropic medication, cortisone and azathioprine were restarted from the beginning. Exactly 19 days after admission, an exanthema appeared on her cleavage and upper back. Simultaneously, the patient reported to suffer from light sensitivity and arthralgia. ANA titer and antibodies against SSA–Ro were rising, C3 complement was low (compare Table 1). Severe skin eruptions were constantly treated in the department of Dermatology, at Ulm University.

In January 2013, the disease fulfilled the criteria of subacute cutaneous lupus erythematosus (psychosis, discoid lesions, photosensitivity, leukopenia, and ANA titer). Despite immediate resumption of immune suppression, the clinical situation was unchanged if not worsening. Because of self injurious behavior she was taken to the protected ward for weeks, partly medical restraint become necessary. Subsequently, instead of azathioprine, mycophenolate mofetil (CellCept^®^ 3 g/day) was prescribed. Nevertheless, there was little therapeutic success to be observed. In March 2013, additionally even the scalp and face were affected by skin lesions. After 10 months of inpatient stay, the patient agreed to another CSF examination, which showed oligoclonal IgG bands (compare Tables 2 and 3). There were no neuronal antibodies. Also levels of tumor necrosis factor (TNF)-α and SCDs 25 were increased in the blood and elevated biomarkers in CSF were found (compare Table 2). The results of CSF examination now justified a diagnosis of NPSLE in accordance with established criteria (12–15).

**Table 2:** Plasma cytokines and CSF cytokines (red framed) of the patient in 2011–2015 based on research done by Prof. Dr. E. Marion Schneider, Experimental Anesthesiology Section, University Clinic Ulm, 89075 Ulm, Germany.

	29.03.2011	18.06.2012	26.06.2012	10.12.2012	12.03.2013	15.03.2013	09.04.2013	23.05.2013	13.06.2013	13.06.2013 (CSF)	21.01.2015	Unit
EPO		5.55	6.39	11.00	7.03	10.20	21.10	3.74	15.50		5.77	mU/ml
IL-10	0.96	0.92	0.47	0.48	2.20	3.85	1.41	0.88	0.00	2.64	6.91	pg/ml
IL-1β	4.40	2.12	3.44	1.85	1.98	0.92	1.68	0.43	38.70	1.08	3.98	pg/ml
IL-6	0.28	0.62	0.215	4.77	3.40	2.33	3.11	0.94	131.00	8.41	1.34	pg/ml
IL-8	16.80	4.63	1.61	7.70	18.20	11.60	6.84	6.45	8.84	53.70	4.47	pg/ml
TNF-α	23.60	8.05	8.90	15.30	18.50	19.60	15.80	9.93	14.90	7.26	24.40	pg/ml
sCD25	588.00	603.00	591.00	421.00	865.00	986.00	1,422.00	1,246.0	1,126.00	1.32	849.00	U/ml
LBP	7.70	5.15	7.11	4.34	11.80	11.80	9.23	9.04	4.06	0.76	3.41	ng/ml
Ferritin	53.20	79.60	77.00	59.20	163.00	160.00	65.00	17.80	14.50	3.23	124.00	ng/ml

*Normal range: erythropoietin (EPO) <30 mU/ml; IL-10 <3–15 pg/ml; IL-1β <1–3 pg/ml; IL-6 <6–10 pg/ml; IL-8 <70.0 pg/l; tumor necrosis factor (TNF)-α <3–15 pg/ml; sCD25 = 200–500 U/ml (adult); plasma ferritin <50 ng/ml; lipopolysaccharide binding protein (LBP) <15 μg*.

**Table 3:** CSF parameters in our patients sample (13 June 2013).

Cells			

CSF leukocytes, 1/μl		CSF erythrocytes, 0/μl	

Proteins			

	CSF	Serum	*Q* (CSF/serum) <10^3^
Total protein	320 mg/l		
Albumin	169.00 mg/l	40.7 g/l	4.2
IgG	26.0 mg/l	11.2 g/l	2.3
IgA	1.77 mg/l	1.63 g/l	1.1
IgM	<0.142 mg/l	0.604 g/l	<0.2

Oligoclonal IgG in CSF with additional identical bands in CSF and serum			

Lactate	1.4 mmol/l

## Overcoming Therapy Resistance with Monoclonal Antibody Therapy

**Figure 2:**
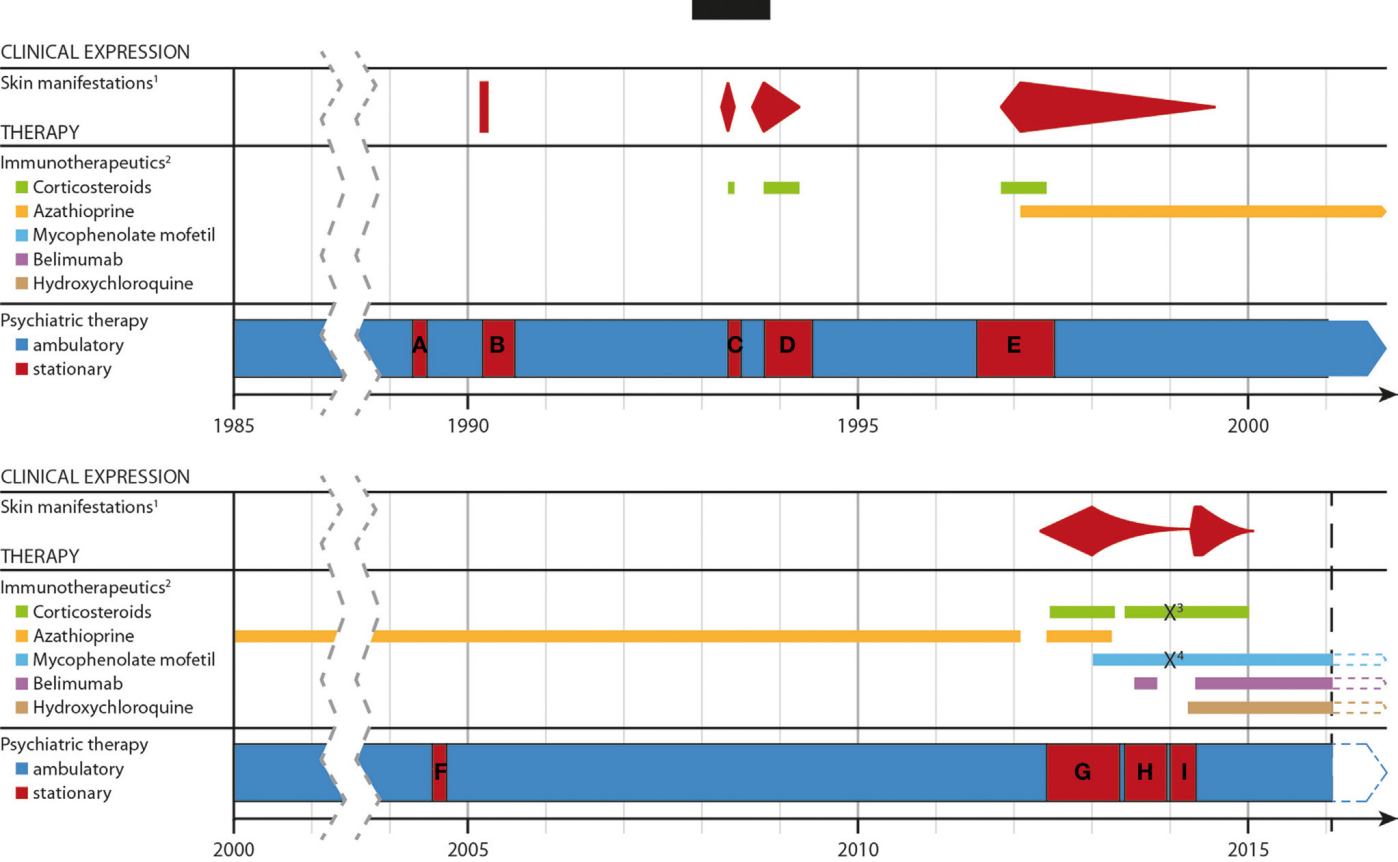
Timeline of the development of the disease, the severity of the skin lesons and immunotherapeutic treatments over time. Abbreviations and explanations: ^1^well documentated, specific cutaneous, and mucosal lesions; ^2^except for topical application; ^3^/^4^methylprednisolone/mycophenolate mofetil was paused due to a suicide attempt. **(A–G)** period of stationary treatment: **(A)** 14.04.–23.06.89; **(B)** 15.02.–11.07.90; **(C)** 11.03.–27.07.90; **(D)** 20.08.93–30.03.94; **(E)** 11.06.96–12.06.97; **(F)** 19.07.–25.09.2004; **(G)** 29.05.12–03.05.13; **(H)** 29.05.–13.12.1; **(I)** 03.01.–30.04.14 and 01.05.–06.05.14.

Because of therapy resistance for more than 1 year and the initial diagnoses of SCLE with NPSLE, actual immune suppressive therapy was reconsidered, and intravenous therapy with the monoclonal antibody belimumab (Benlysta^®^) was started as an add-on treatment to the previous therapy (see Figure 2). Gradually, the patient improved over the next 2 months in every aspect and was discharged in December 2013. However, 2 weeks later she again attempted suicide by drug intoxication, requiring treatment in the intensive care unit, where all psychiatric and immune suppressive treatments were stopped. Eight weeks later, severe skin eruptions again emerged. In spite of hydroxychloroquine (Quensyl^®^ 400 mg/day) being prescribed now, the skin eruptions worsened further, and psychiatric hospitalization was again required because of her severe psychosis relapse. Subsequently belimumab was restarted again. With the combination of mycophenolate mofetil (1,000 mg/day), hydroxychloroquine (400 mg/day), urbason (2 mg/day), and belimumab (10 mg kg/bodyweight per cycle) a rapid remission was achieved. Both, psychotic symptoms and skin manifestations improved in parallel. An attempt at pausing hydroxychloroquine was discarded because of repeated erupting skin manifestations. The patient was discharged from hospital treatment in an acceptable clinical status. During the next continuous outpatient treatment months she improved to full remission in psychosis and skin manifestations.

Following dismissal, she remained clinically stable with a complex of psychopharmacological and immune suppressive medications (for details see Figure 2).

## Discussion

We conducted a detailed analysis case study of a patient with schizophrenia in regard to the question of whether autoimmunity and schizophrenia were unrelated comorbid disorders, or whether both disorders could be interpreted as a unified disorder, representing in fact the varied manifestations of an atypical course of SLE, predominantly with psychiatric symptoms and accompanying skin manifestations most over the time. The latter hypothesis was plausible from a first look because of concurrent minor autoimmune signs and findings with the psychotic phases. To assume indeed a unifying disease hypothesis, we need, however, more arguments. The following points support the unifying perspective:

1. Good clinical improvement of skin and psyche under short time corticosteroids in 1993 with repeated relapses after corticosteroids was discontinued.
2. Repeated temporal parallelism of the appearance of skin manifestations and psychiatric manifestations consistently over the disease course of 25 years.
3. Some temporal relationship between high titer of autoimmune/inflammatory markers and antinuclear antibodies to the severity of psychiatric symptoms.
4. Striking temporal parallelism of improvement of most severe psychiatric and skin manifestations with complex immune suppression during the last severe relapse.
5. Close temporal relationship between longtime full remission under azathioprine medication, followed by the most severe relapse ever after discontinuance. In addition, there is a plausible delay of a few weeks after discontinuance of azathioprine and onset of first psychotic symptoms.
6. Demonstration of mild neuroinflammation by CSF analysis during the most severe disease phase, when fulfilling criteria of SCLE, fulfilling thus criteria of NPSLE, the latter appearing as an acute severe schizophreniform psychosis.
7. Suspicious family history with both severe psychiatric [depression in both parents (ICD F 33.3); sister with depression and paranoid schizophrenia (ICD-10 F20.0, F32.2)] and autoimmune disorders [sister with Crohn’s disease (ICD-10 K50.1)].
8. Unnoticed autoimmune signs were retrospectively assessed with a newly developed questionnaire [details in Ref. (16), in review], yet detected in the prepsychotic phase repeated arthralgia and swelling of limbs and fingers, which happened in our patient.
9. Clinical improvement of psychosis repeatedly seemed associated with immune or anti-inflammatory therapies, including lithium with immune suppressive reactions.
10. Temporal relationship of increasing serum cytokines, especially TNF-α, during the most severe psychotic relapse and uniquely elevated CSF cytokines fitting to observations in schizophrenic patients (see Table 2; see below).

Because our patient rejected repeatedly CSF diagnostics, there is only a single CSF sample with cytokine profiling. The sampling was done in June 2013, during a phase of severe psychotic symptoms. In this CSF sample, we found suspicious biomarkers (compare Table 2): chemokine IL-8 was selectively higher than in the corresponding serum samples, other CSF cytokines were marginally elevated, TNF-α was not increased. These results match with CSF and serum cytokine profiling in schizophrenic and affective spectrum disorder patients (7). The presence of oligoclonal bands in CSF, few of them also in serum (compare Table 3), indicates in agreement with established criteria (13, 14) neuroinflammation, here overall mild and such was found in small groups of patients with affective and schizophrenic spectrum disorders in several studies (5, 6).

We conclude from our case analysis of 25 years disease course, that our case represented an atypical case of autoimmune disorder, lately diagnosed as LE, beginning with a focus on the CNS, explaining the variant psychiatric Syndrome from variant autoimmune-inflammatory disease activity. We could only partially define the apparently variant disease activity and only partially detect the immune inflammatory pathomechanisms involved. Overall the case represents a prototype of what was preliminary defined and hypothesized as ME. Cases with incomplete diagnostic autoimmune disorders over years, which later may develop a classified autoimmune disorder, are well-known, for example, presenting with mixed tissue disorder years before (17–20). Comparable cases of NPSLE were not described according to our knowledge. Our many arguments to assume an atypical case of remitting relapsing, or chronic when without immune suppressive treatment, NPSLE may be strong, though proof is missing: there were no definite, in part from circumstantial reasons, signs of neuroinflammation by imaging or CSF analysis in the early course. However, such was not unexpected from many arguments (9) and in rare single cases of schizophrenic syndromes with normal or quite normal CSF, nevertheless brain biopsy demonstrated mild inflammation (11, 21). We did not find neuronal antibodies, but this does not argue against our conclusion, cases with brain biopsy proven ME described by Najjar et al. (11) did also not demonstrate neuronal autoantibodies. We had not the opportunity to take a brain biopsy. On the other hand, we had the advantage of a long-term observation including several time periods of immune suppressive treatment, given from somewhat only partially established diagnostic criteria, detecting in retrospect a rather plausible if not clear evidence of positive treatment effects from immune suppression, which continues up to the time point of writing this paper. In sum, our arguments appear to represent strong arguments in total, to interpret our case as an atypical course of NPSLE from the beginning focused on the CNS.

With this case several difficult scientific and ethical questions had to be considered: After remission of the debated severe skin disease, thought to represent an allergic reaction to pharmaca or an undefined autoimmune disorder, also the possibility of a unified autoimmune disorder, as discussed in this paper, was considered by author Karl Bechter and discussed with the patient, though by majority of psychiatric doctors in our hospital rather held implausible. The patient was clearly informed about these scientific uncertainties and the novelty of the hypothesis of mild neuroinflammation to possibly causally underlie the psychotic disorder, held by Karl Bechter. The question of a relative indication of long-term treatment with azathioprine was evaluated independently by specialist in internal medicine (Dr. Peter Müller), with the result that long time azathioprine was justified (defined as relative indication) by the case history and because of continued mild leukopenia. With this informed knowledge the patient preferred to be treated with azathioprine as documented and the psychopharmacological medication to be reduced. The patient continued to visit Drs. Karl Bechter and Peter Müller, all over the respective years reported, though visiting Karl Bechter only sporadically but continuous over many years with remitted psychiatric syndrome but immediately with incipient relapse after set off of azathioprine.

## Difficulties in Diagnosing Autoimmune Disorders in Psychiatric Patients

Psychiatric symptoms are rarely reported as an initial and isolated feature of SLE (22), in spite of many patients having the feeling that psychiatric symptoms occurred before they were diagnosed with SLE. However, central inflammation does not always coincide with systemic signs. Tests on mice suggest that NPSLE is not always a complication of SLE and can occur in absence of systemic autoimmunity (23). This supports the importance that early CSF diagnostics are done in longtime psychiatric patients. The European League Against Rheumatism emphasizes the increased risk of neuropsychiatric manifestations in SLE and requests early diagnostics including CSF examination in neuropsychiatric patients (even if only to exclude CNS infections) (24).

In our case, there was a delay of 4 years between initial psychiatric symptoms and first noticed mild skin manifestations. Accompanying systemic signs are maybe misinterpreted or remain undiagnosed, in our case even the established criteria of lupus were fulfilled only late in the course. This case shows the difficulties in establishing a diagnosis of common autoimmune disorders such as SLE, by regarding criteria like those of the American College of Rheumatology (ACR)-97 or Systemic Lupus International Collaborating Criteria (SLICC)-12. Our patient never had any further neuropsychiatric symptoms like seizures or neuropathy. No cerebrovascular accidents were noticed. Missing manifestations or incomplete laboratory markers create major uncertainties for clinicians. The general problem of incomplete criteria is known in autoimmune disorders, the diagnosis of mixed tissue disorder, a respective phenomenon, number of cases later being diagnosed with classical autoimmune disorders (19). Also atypical courses in other organ systems, e.g., with visceral focus have been described (25).

## Conclusion

Psychosis as the initial or predominant manifestation of SLE over years is an unusual course of the disease presented here. If our conclusion is correct, that indeed we observed one disorder with manifestations in different organ systems, there is an apparent need for improved diagnostic instruments and methods. For better detection of unnoticed clinical signs we recently developed a questionnaire called “FAISF” [(16), in review]. Another problem is, as our case strikingly demonstrates, that even known signs and minor findings of autoimmunity do not usually allow a definitive diagnosis of the defined autoimmune disorder, as established for example by the ACR or the SLICC. This problem of sub diagnostic cases has also been recognized in rheumatology.

A careful analysis of our case with 25 years disease including actual and previous material, clearly suggests that this case of seemingly comorbid schizophrenia with minor autoimmunity signs represented a case of one emerging autoimmune disorder with variant manifestations systemically and within the CNS, though atypically with predominant appearance as a schizophrenia spectrum disorder. We do not exclude, that more such cases exist, but were certainly inappropriately understood. This case demonstrates the therapeutic potential if detected in an early phase and treated appropriately including with immunosuppressive options. The consequences of our perspective would likely include psychoimmunological aspects in psychiatric routine examinations. Recommendations should be developed including clinical signs and findings.

## Author Contributions

AM—collecting data and summary of the patient. CP and KB—treatment of the patient. EMS—CSF analysis, serum analysis, and cytokine profiling.

## Conflict of Interest Statement

The authors declare that the research was conducted in the absence of any commercial or financial relationships that could be construed as a potential conflict of interest.
